# Sites of Peripheral Artery Occlusive Disease as a Predictor for All-Cause and Cardiovascular Mortality in Chronic Hemodialysis

**DOI:** 10.1371/journal.pone.0128968

**Published:** 2015-06-02

**Authors:** Ming-Hsien Tsai, Hung-Hsiang Liou, Jyh-Gang Leu, Ming-Fang Yen, Hsiu-Hsi Chen

**Affiliations:** 1 Division of Nephrology, Department of Internal Medicine, Shin-Kong Wu Ho-Su Memorial Hospital, Taipei, Taiwan; 2 Division of Nephrology, Department of Medicine, Hsin-Jen Hospital, New Taipei City, Taiwan; 3 School of Oral Hygiene, College of Oral Medicine, Taipei Medical University, Taipei, Taiwan; 4 Division of biostatistics, Institutes of Epidemiology and Preventive Medicine, College of Public Health, National Taiwan University, Taipei, Taiwan; Nagoya University, JAPAN

## Abstract

**Background:**

The ankle—brachial blood pressure (BP) index (ABI) not only indicates the presence of peripheral artery occlusive disease (PAOD) but predicts mortality in patients undergoing hemodialysis (HD). However, whether the site of PAOD can provide additional contribution to predicting mortality have not been investigated yet. Our primary objective was to determine the associations between the site of PAOD and all-cause and cardiovascular mortality in chronic HD (CHD) patients.

**Methods:**

A retrospective cohort study was conducted to evaluate 444 Taiwanese CHD patients between December 2006 and June 2013. The site of PAOD together with other explanatory variables such as demographic data, body mass index, a history of cardiovascular diseases, HD vintage, biochemical data, and cardiothoracic ratio (CTR) were assessed by the Cox proportional hazards regression model.

**Results:**

The frequency of PAOD was 14.6% in both legs, 4.9% in the right side only, and 5.1% in the left side only. During the study period, 127 all-cause and 93 cardiovascular deaths occurred. PAOD site was found to have significant predictive power for all-cause mortality with the order of 3.04 (95% CI: 1.56–5.90) hazard ratio on the right side, 2.48 (95% CI: 1.27–4.82) on the left side, and 4.11 (95% CI: 2.76–6.13) on both sides. The corresponding figures for cardiovascular mortality were 3.81 (95% CI: 1.87–7.76) on the right side, 2.76 (95% CI: 1.30–5.82) on the left side, and 3.95 (95% CI: 2.45–6.36) on both sides. After adjustment for other explanatory variables, only right-sided PAOD still remained to have significant predictive power for all-cause and cardiovascular mortality and bilateral PAOD kept the significant association with all-cause mortality.

**Conclusions:**

The site of PAOD revealed various predictive powers for all-cause and cardiovascular mortality in CHD patients and only right-sided PAOD remained an independent predictor for both types of mortality making allowance for relevant confounding factors.

## Introduction

The ankle—brachial index (ABI; the ratio of ankle to brachial systolic blood pressure [BP]) has been proven to be a simple, noninvasive, and reliable tool for the diagnosis of PAOD and a representative of systemic vascular disease. A significant decrease in patients with end-stage renal disease (ESRD) is attributed to the common co-morbid cardiovascular disease (CVD)[1–3]. Identifying the risk factors responsible for all-cause and cardiovascular (CV) mortality may present an opportunity for early intervention and the improvement of survival in these patients. Peripheral artery occlusive disease (PAOD), an atherosclerotic disorder, is frequently found in CKD patients [4, 5] and HD patients [6, 7], and has been demonstrated as a predictor for all-cause and cardiovascular mortality [7–9].

Patients are considered with PAOD when their either leg shows a lower ABI (<0.9). However, the severity of PAOD with an ABI value < 0.9 in clinical practice[10, 11] may vary with each leg. Further evidence also shows that a blood pressure difference between the legs (a difference in systolic BP of ≥15 mmHg or diastolic BP of ≥10 mmHg) was relevant to PAOD and was significantly associated with overall and CV death in HD patients [12]. The unequal limb atherosclerosis had an association with the interleg BP difference [8, 13]. Lin et al. had used the interleg ABI difference to make the limb atherosclerosis difference comparable between individuals and verified that an interleg ABI difference of ≥0.15 is an independent risk factor for all-cause mortality in patients with CHD [14]. In spite of those findings, there has been limited data about various sites of PAOD in association with all-cause and CV mortality. The aim of this study was to investigate whether PAOD site is an independent predictor for all-cause and CV mortality with adjustments for other explanatory factors in patients undergoing CHD in Taiwan.

## Study Population and Methods

### Study Design and Patients

Patients were included in to our study between December 2006 and January 2009. The inclusion criterion was at least 3 months of regular HD in the dialysis unit of Shin Kong Wu Ho-Su Memorial Hospital. The observation continued until June 2013. We excluded patients with any acute cardiovascular, cerebrovascular, infectious, or other active diseases in 3 months before entering this study. Patients with ABI values >1.3 were also excluded from analysis.

### History collection and laboratory data

Demographic and medical data were obtained from the patients’ medical records at entry to the study (baseline). The following data were included: age, gender, body mass index (BMI, weight/height^2^), cardiothoracic ratio (CTR), smoking (ever versus never), comorbid conditions, serum creatinine, albumin, lipid profiles, iron profiles, hemoglobin (Hb), intact parathyroid hormone (iPTH), ionized calcium (iCa), phosphate (P), and dialysis efficiency (Kt/V). Moreover, we also collected the baseline medication history, including anti-platelet, renin-angiotensin system (RAS) blockade, beta-blocker, and statin. CVD was diagnosed using documented histories of coronary artery disease or cerebrovascular disease. Patients with CTR > 0.5 on chest radiographs were diagnosed with cardiomegaly. Blood specimens were collected after at least 8 h of fasting before dialysis sessions. Kt/V was determined according to the procedure described by Shinzato *et al* [15].

### Ethic Statements

The Institutional Review Boards of the Shin-Kong Wu Ho-Su Memorial Hospital, Taipei, Taiwan, approved the study and waived the informed consent because our study was base on medical chart review. The patients’ information were anonymized and de-identified prior to analysis.

### ABI measurement

The ABIs were measured with an Oscillometric machine (VaSera VS-1000; Fukuda Denshi, Tokyo, Japan), with simultaneous BP measurements in the arm without vascular access and around both ankles. The systolic pressure of the arm without dialysis access and the bilateral ankle pressures were used separately for the calculations. ABI was calculated automatically by dividing the ankle pressure by the brachial pressure. The ABI measurement was performed once for each patient before a hemodialysis session. Patients with ABI < 0.9 were diagnosed with PAOD, implying varying degrees of atherosclerosis in the lower extremities. We also defined right-sided PAOD for ABI <0.9 in only the right leg and left-sided PAOD for ABI <0.9 in only the left leg. Moreover, right-sided dominance of ABI represented a lower ABI value in right leg than left leg and vice versa.

### Statistical analyses

Patients were lost to follow-up if a change in HD units was documented, and their data were censored at the date of the last documented contact in our dialysis unit. The outcome measures were all-cause and CV mortality (ascertained from Shin Kong Wu Ho-Su Memorial Hospital electronic records). Data are expressed as mean ± standard deviation (SD. The Kruskal—Wallis test or Mann—Whitney U test was used to compare the means of continuous variables and the χ^*2*^ test was used for categorical variables. Survival curves were estimated by using the Kaplan—Meier method and tested with the log-rank test. Moreover, crude and multi-variable analyses of mortality were performed using a Cox proportional hazards model to determine the hazard rate of death as a function of the different sites of PAOD. The assumption of proportionality was not violated, which was tested by using time-dependent Cox regression model. The adjusted confounders in multivariable model included all parameters, which are significant in univariate mode and those parameters showing significant difference between groups. A *P*-value of ≤0.05 was considered statistically significant. All statistical analyses were performed using the statistical package for social sciences statistical software (SPSS version 18; IBM Inc, Chicago, IL, USA).

## Results

### Patient background information

A total of 444 ESRD patients who received regular HD sessions with a dialysis vintage of 7.7 ± 5.0 years were enrolled. The mean age of the cohort was 61.6 ± 13.1 years (Interquartile range, 53–71 years), and the mean follow-up duration was 51.5 ± 21.9 months. During the follow-up period, 127 (28.6%) all-cause mortality events were ascertained in these patients, including fatal CV events (*n* = 93), malignancies (*n* = 9), infectious diseases (*n* = 17), gastrointestinal bleeding (*n* = 2), and others (*n* = 6). Eighty patients dropped out of the study. Table 1 shows the baseline characteristics of the study population. We classified the participants into four subgroups based on the presence and site of PAOD (non-PAOD, right-sided PAOD, left-sided PAOD, and bilateral PAOD). Table 2 shows the baseline characteristics of patients stratified by site. The prevalence of bilateral PAOD was 14.6% (65/444), of right-sided disease was 4.9% (22/444), and of left-sided disease was 5.1% (23/444). The difference across the four subgroups was statistically significant with respect to age (*P* < 0.001), gender (*P* = 0.05), presence of DM (*P* < 0.001), previous CVD (*P* = 0.040), systolic and diastolic BP (*P* < 0.001), serum albumin (*P* < 0.001), triglycerides (*P* = 0.021), total cholesterol (*P* = 0.028), Kt/V (*P* = 0.043), and cardiomegaly (*P* = 0.09). In the comparison between patients with PAOD, there was significant difference in age, gender, DM history, serum albumin, cholesterol, and calcium-phosphate product (all *P*<0.05). Right-sided PAOD had a lower age, presence of DM, and Kt/V; a higher calcium—phosphate product and nutrition status (serum albumin and cholesterol level) than did left-sided and bilateral PAOD.

**Table 1:** Characteristics of study population.

Characteristic	All patients (444)
Age (years)	61.6 ± 13.1
Males (%)	206 (46.4)
Duration of dialysis (year)	7.7 ± 5.0
Diabetes mellitus (%)	145 (32.7)
Previous CVD (%)	93 (20.9)
Smoking (%)	92 (20.7)
Systolic BP (mmHg)	151 ± 77
Diastolic BP (mmHg)	78 ± 17
Body mass index (kg/m2)	22.9 ± 3.7
Albumin level (g/dL)	4.1 ± 0.3
Triglyceride (mg/dL)	152 ± 124
Cholesterol level (mg/dL)	175 ± 42
Kt/V	1.66 ± 0.23
Cardiothoracic ratio	0.5 ± 0.07
Hemoglobin (g/dL)	10.4 ± 1.3
iPTH (pg/mL)	193 ± 204
Ferritin (μg/dL)	636 ± 784
Ca P product (mg/dL)2	48.2 ± 13.5
Right side ABI	1.03 ± 0.18
Left side ABI	1.03 ± 0.20
Medications	
Anti-platelet use (%)	154 (34.7)
RAS blockader use (%)	90 (20.3)
Beta-blocker use (%)	142 (32.0)
Statin use (%)	143 (32.2)
All cause mortality events (%)	127 (28.6)
Cardiovascular mortality events (%)	93 (20.9)

Abbreviations: CVD, cardiovascular disease; BP, blood pressure; iPTH, intact parathyroid hormone; Ca, calcium; P, phosphate; ABI, ankle-brachial index; RAS, renin-angiotensin system.

**Table 2:** Characteristics of patients at inclusion according to the location of PAOD.

Characteristic	Non (*n* = 334)	Right (*n* = 22)	Left (*n* = 23)	Bilateral (*n* = 65)	*P* *	*P* ^#^
Age (years)	59.3 ± 13.1	65.0 ± 10.9	73.0 ± 7.9	68.6 ± 10.6	<0.001	0.017
Male (%)	165 (49)	12 (54)	12 (52)	17 (26)	0.005	0.015
Duration of dialysis (years)	7.8 ± 5.1	6.7 ± 4.6	8.5 ± 5.6	6.9 ± 4.3	0.526	0.559
Diabetes mellitus (%)	83 (24)	8 (36)	10 (43)	44 (67)	<0.001	0.014
Previous CVD (%)	63 (18)	5 (22)	3 (13)	22 (34)	0.040	0.136
Smoking history (%)	75 (22)	5 (22)	5 (21)	7 (10)	0.204	0.262
Systolic BP (mmHg)	161 ± 82	141 ± 44	114 ± 30	116 ± 44	<0.001	0.077
Diastolic BP (mmHg)	82 ± 15	74 ± 17	62 ± 11	64 ± 19	<0.001	0.081
Body mass index (kg/m2)	22.9 ± 3.8	22.7 ± 2.7	22.0 ± 3.3	23.2 ± 4.0	0.624	0.396
Albumin level (g/dL)	4.2 ± 0.3	4.2 ± 0.4	4.0 ± 0.3	3.9 ± 0.3	<0.001	0.008
Triglyceride (mg/dL)	147 ± 126	209 ± 185	133 ± 79	167 ± 95	0.021	0.278
Cholesterol level (mg/dL)	173 ± 40	207 ± 65	159 ± 40	179 ± 38	0.028	0.030
Kt/V	1.65 ± 0.24	1.58 ± 0.18	1.73 ± 0.21	1.71 ± 0.20	0.043	0.056
Cardiomegaly (%)	142 (42)	9 (40)	13 (56)	41 (63)	0.009	0.138
Hemoglobin (g/dL)	10.5 ± 1.4	10.6 ± 1.7	10.2 ± 1.3	10.2 ± 1.1	0.281	0.496
iPTH (pg/mL)	202 ± 211	191 ± 170	136 ± 118	166 ± 196	0.109	0.528
Ferritin (μg/dL)	648 ± 893	556 ± 230	597 ± 189	620 ± 202	0.784	0.660
Ca P product (mg/dL)2	48.8 ± 13.7	54.2 ± 12.7	42.8 ± 10.8	44.5 ± 12.8	0.003	0.004
Medications						
Anti-platelet use (%)	123 (36)	12 (54)	16 (69)	51 (78)	0.074	0.095
RAS blockade use (%)	72 (21)	3 (13)	6 (26)	9 (13)	0.379	0.366
Beta-blocker use (%)	105 (31)	10 (45)	7 (30)	20 (30)	0.583	0.423
Statin use (%)	103 (30)	8 (40)	11 (47)	21 (32)	0.387	0.413

Abbreviations: CVD, cardiovascular disease; BP, blood pressure; iPTH, intact parathyroid hormone; Ca, calcium; P, phosphate; RAS, renin-angiotensin system

*Comparison between all four groups

^#^ Comparison between all groups except non-PAOD group.

### Kaplan—Meier survival curves

**Fig 1:**
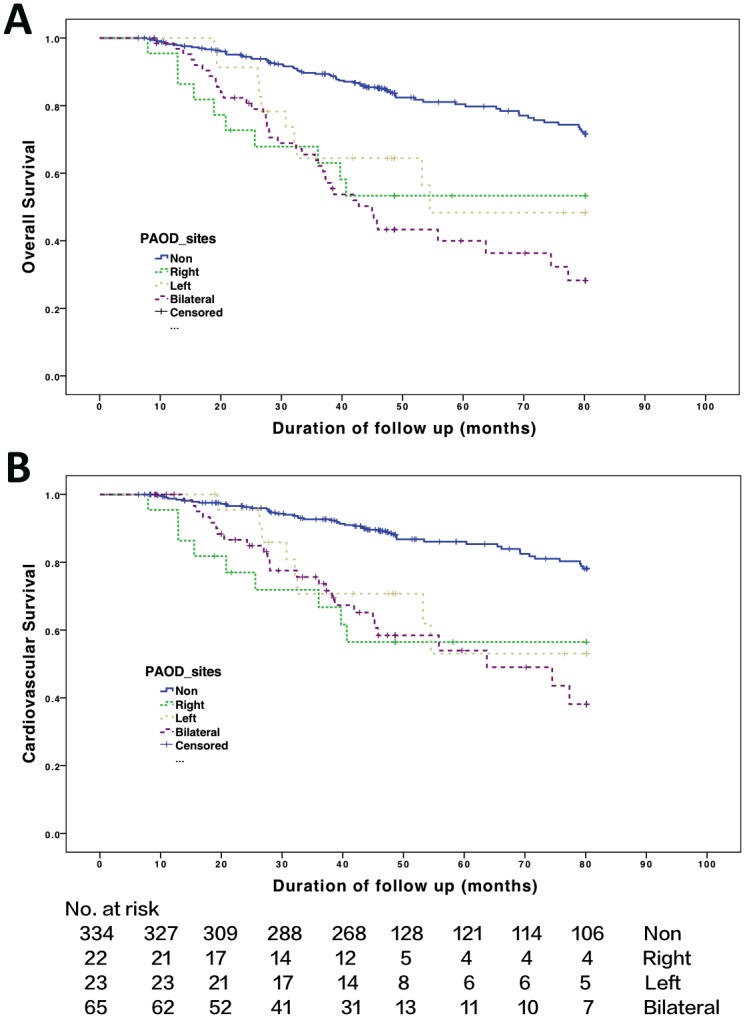
Kaplan—Meier survival curves. Probabilities of (A) overall survival with log-rank test: χ^2^ = 60.89; *P* ≤ 0.001 in the four groups and χ^2^ = 2.02; *P* = 0.364 in the three PAOD groups. (B) Cardiovascular survival with log-rank test: χ^2^ = 45.24; *P* ≤ 0.001 in the four groups and χ^2^ = 0.69; *P* = 0.708 in the three PAOD groups.

Fig 1 shows that the Kaplan—Meier survival curves of all-cause (Fig 1A) and CV mortality (Fig 1B) according to the site of PAOD. The difference in survival among the four groups was significant for all-cause (χ^2^ = 60.89, *P* < 0.001) and CV (χ^2^ = 45.24, *P* < 0.001) mortality using the log-rank test. However, no significant difference in survival among the three groups of right side, left side, and bilateral PAOD was noted.

### All-cause mortality in CHD patients

During the 6.6-year follow-up period, 127 deaths were recorded. Compared with patients without PAOD, the hazard ratio (HR) for all-cause death was 3.04 (95% CI: 1.56 to 5.90) in patients with right-sided PAOD, 2.48 (95% CI: 1.27 to 4.82) with left-sided PAOD, and 4.11, (95% CI: 2.76 to 6.13) with bilateral PAOD (Table 3). Other significant predictors for all-cause death included age, DM, previous CVD, systolic and diastolic BP, serum albumin, cardiomegaly, and calcium phosphate products (all *P* < 0.05). In the multi-variable analysis, the HR for all-cause death was 3.04 (95% CI: 1.50 to 6.14) in patients with right-sided PAOD, 1.36 (95% CI: 0.65 to 2.82) with left-sided PAOD, and 1.86 (95% CI: 1.09 to 3.16) for bilateral PAOD after adjustment for age, gender, DM, previous CVD, Kt/V, CTR> 0.5, blood pressure, statin use and serum albumin, triglycerides, cholesterol, and calcium—phosphate product. Among the risk factors, HRs for age (1.02, 95% CI: 1.00–1.04), previous CVD (1.92, 95% CI: 1.29–2.84), cardiomegaly (1.63, 95% CI: 1.10–2.41), and serum albumin level (0.25, 95% CI: 0.14–0.44) remained significant.

**Table 3:** Cox proportional hazards regression analysis for all-cause mortality.

	Crude		Multivariable	
Parameter	Hazard ratios (95% CI)	P	Hazard ratios (95% CI)	P
Age (per year)	1.05 (1.03–1.06)	<0.001	1.02 (1.00–1.04)	0.005
Male versus female	1.04 (0.73–1.47)	0.814	1.47 (0.94–2.28)	0.086
Duration of dialysis (per year)	0.99 (0.95–1.03)	0.727		
Diabetes mellitus	1.92 (1.35–2.72)	<0.001	1.07 (0.69–1.65)	0.738
Previous CVD	2.41 (1.66–3.48)	<0.001	1.92 (1.29–2.84)	0.001
Smoking (ever versus never)	1.10 (0.72–1.67)	0.645		
Systolic BP (per 1 mmHg)	0.98 (0.98–0.99)	<0.001	1.00 (0.99–1.00)	0.908
Diastolic BP (per 1 mmHg)	0.97 (0.96–0.98)	<0.001	0.99 (0.99–1.00)	0.323
Body mass index (per 1 kg/m2)	0.98 (0.93–1.03)	0.461		
Albumin level (per 1 g/dL)	0.18 (0.12–0.28)	<0.001	0.25 (0.14–0.44)	<0.001
Triglyceride (per 1 mg/dL)	0.99 (0.99–1.00)	0.100	0.99 (0.99–1.00)	0.065
Cholesterol level (per 1 mg/dL)	0.99 (0.99–1.00)	0.329	1.00 (0.99–1.01)	0.233
Kt/V (per 1 unit)	0.82 (0.38–1.76)	0.628	1.05 (0.40–2.74)	0.914
Cardiomegaly	2.33 (1.62–3.36)	<0.001	1.63 (1.10–2.41)	0.015
Hemoglobin (per 1 g/dL)	0.89 (0.78–1.02)	0.102		
iPTH (per 1 pg/mL)	0.99 (0.99–1.00)	0.244		
Ferritin (per 1.0 μg/dL)	1.00 (0.99–1.00)	0.558		
Ca P product (per 1 mg2/dL2)	0.98 (0.97–0.36)	0.017	1.00 (0.98–1.01)	0.590
Medications				
Anti-platelet use	0.87 (0.60–1.27)	0.481		
RAS blockade use	1.14 (0.75–1.73)	0.514		
Beta-blocker use	0.72 (0.48–1.08)	0.726		
Statin use	0.64 (0.42–0.96)	0.031	0.691 (0.45–1.05)	0.086
PAOD (versus non)				
Right side	3.04 (1.56–5.90)	0.001	3.04 (1.50–6.14)	0.002
Left side	2.48 (1.27–4.82)	0.007	1.36 (0.65–2.82)	0.407
Bilateral	4.11 (2.76–6.13)	<0.001	1.86 (1.09–3.16)	0.022

Abbreviation: CVD, cardiovascular disease; BP, blood pressure; RAS, renin-angiotensin system; iPTH, intact parathyroid hormone; Ca, calcium; P, phosphate; PAOD, peripheral arterial occlusion disease.

### Cardiovascular mortality in CHD patients

Ninety-three fatal CV events were ascertained during the follow-up period. Table 4 shows the results of the Cox proportional hazards regression analysis of various parameters as predictors for CV mortality. Compared with patients without PAOD, the crude HRs for CV mortality in patients with right-sided PAOD, left-sided PAOD, and bilateral PAOD were 3.81 (95% CI: 1.87–7.76), 2.76 (95% CI: 1.30–5.82), and 3.95 (95% CI: 2.45–6.36), respectively. Other significant predictors in the univariate analysis were age (1.04, 95% CI: 1.02–1.06), a history of DM (2.02, 95% CI: 1.34–3.05), previous CVD (2.26, 95% CI: 1.46–3.49), systolic BP (0.98, 95% CI: 0.98–0.99), diastolic BP (0.96, 95% CI: 0.95–0.97), albumin level (0.19, 95% CI: 0.12–0.32), and cardiomegaly (2.22, 95% CI: 1.45–3.39). After adjusting for a history of cardiomegaly, previous CVD, low serum albumin level, and advanced age in the multi-variable regression model, right-sided PAOD (3.65, 95% CI: 1.72–7.74) was still significant for CV mortality, whereas left-sided PAOD (1.11, 95% CI: 0.48–2.53) and bilateral PAOD (1.51, 95% CI: 0.79–2.87) were no longer statistically significant.

**Table 4:** Cox proportional hazards regression analysis for cardiovascular mortality.

	Crude		Multivariable	
Parameter	Hazard ratios (95% CI)	*P*	Hazard ratios (95% CI)	*P*
Age (per 1 year)	1.04 (1.02–1.06)	<0.001	1.02 (1.00–1.04)	0.042
Male versus female	1.13 (0.75–1.70)	0.541	1.87 (1.11–3.16)	0.018
Duration of dialysis (per 1 year)	1.00 (0.95–1.04)	0.983		
Diabetes mellitus	2.02 (1.34–3.05)	0.001	1.20 (0.73–1.98)	0.463
Previous CVD	2.26 (1.46–3.49)	<0.001	1.91 (1.20–3.04)	0.006
Smoking (ever versus never)	1.27 (0.79–2.04)	0.309		
Systolic BP (per 1 mmHg)	0.98 (0.98–0.99)	<0.001	1.00 (0.99–1.00)	0.980
Diastolic BP (per 1 mmHg)	0.96 (0.95–0.97)	<0.001	0.97 (0.95–0.99)	0.041
Body mass index (per 1 kg/m2)	0.96 (0.90–1.02)	0.202		
Albumin level (per 1g/dL)	0.19 (0.12–0.32)	<0.001	0.27 (0.14–0.52)	<0.001
Triglyceride (per 1 mg/dL)	0.99 (0.99–1.00)	0.170	0.99 (0.99–1.00)	0.120
Cholesterol level (per 1 mg/dL)	0.99 (0.99–1.00)	0.409	1.00 (0.99–1.00)	0.334
Kt/V (per 1.0)	0.97 (0.40–2.36)	0.957	1.73 (0.56–5.33)	0.334
Cardiomegaly	2.22 (1.45–3.39)	<0.001	1.58 (1.00–2.50)	0.047
Hemoglobin (per 1 g/dL)	0.86 (0.74–1.01)	0.079		
iPTH (per 1 pg/mL)	1.00 (0.99–1.00)	0.909		
Ferritin (per 1 μg/dL)	1.00 (0.99–1.00)	0.636		
Ca P product (per 1 mg2/dL2)	0.99 (0.97–1.00)	0.178	1.01 (0.99–1.02)	0.240
Medications				
Anti-platelet use	0.95 (0.63–1.47)	0.854		
RAS blockade use	1.06 (0.64–1.73)	0.817		
Beta-blocker use	0.71 (0.45–1.14)	0.163		
Statin use	0.65 (0.40–1.04)	0.075		
PAOD (versus non)				
Right side	3.81 (1.87–7.76)	<0.001	3.65 (1.72–7.74)	0.001
Left side	2.76 (1.30–5.82)	0.008	1.11 (0.48–2.53)	0.800
Bilateral	3.95 (2.45–6.36)	<0.001	1.51 (0.79–2.87)	0.207

Abbreviation: CVD, cardiovascular disease; BP, blood pressure; RAS, renin-angiotensin system; PAOD, iPTH, intact parathyroid hormone; Ca, calcium; P, phosphate; peripheral arterial occlusion disease.

### Right-sided dominance vs. left-sided dominance of ABI

**Fig 2:**
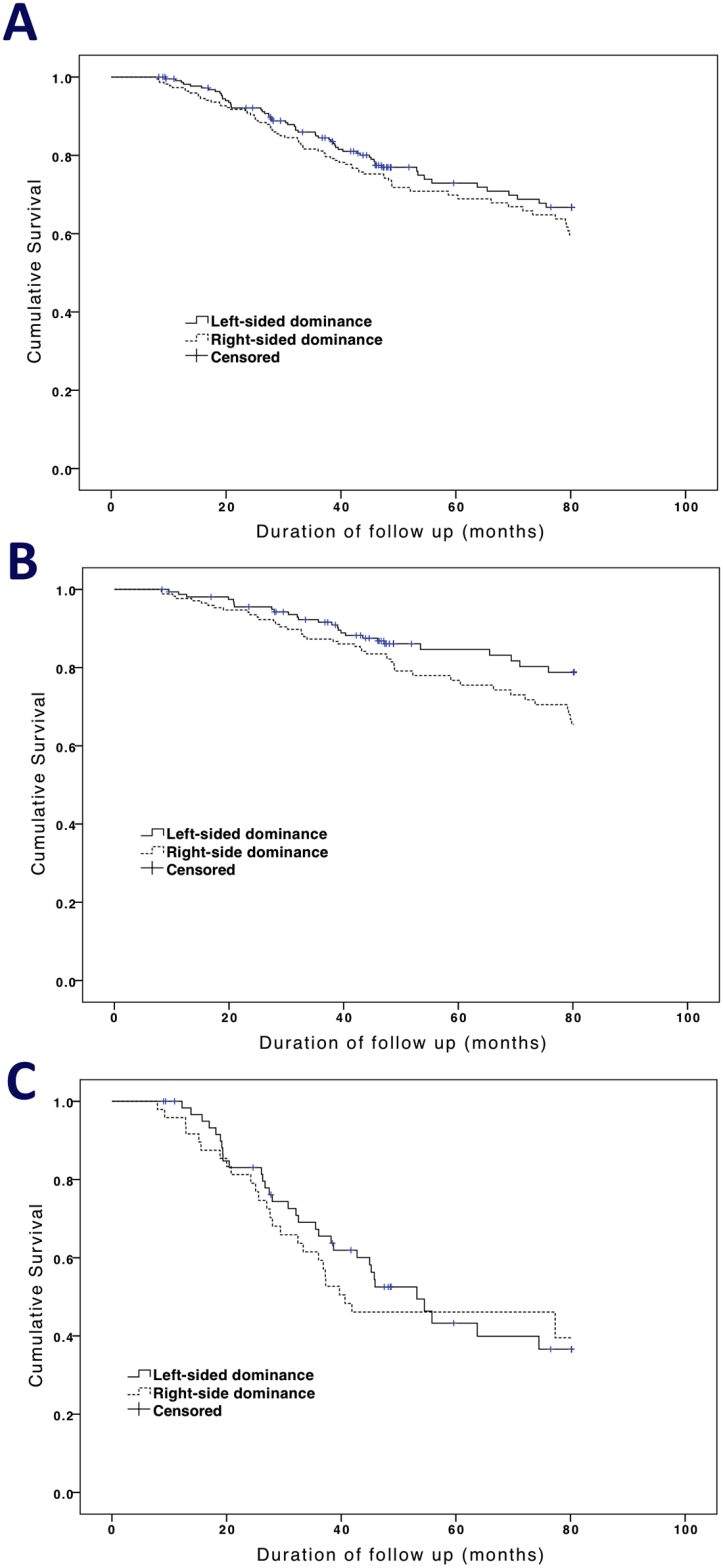
Probabilities of overall survival according to dominance side of ABI value. (A) in all patients with log-rank test: χ^2^ = 1.32; *P* = 0.249; (B) in patients without PAOD with log-rank test: χ^2^ = 3.47; *P* = 0.062; (C) in patients with PAOD with log-rank test: χ^2^ = 0.20; *P* = 0.651.

The prevalence of right-sided dominance in ABI was 50.2% (223/444) and of left-sided dominance was 43.2% (192/444). There was no significant difference of ABI value between legs in individuals (*P* = 0.412, by Wilcoxon signed-rank test). The comparison of survival outcomes between right-sided and left-sided dominance in ABI values is shown in Fig 2, which demonstrated a lacking of significant difference in survival between right-sided and left-sided dominance for ABI values in all patients (Fig 2A, χ^2^ = 1.32, *P* = 0.249), patients without PAOD (Fig 2B, χ^2^ = 3.47, *P* = 0.062), and patients with PAOD (Fig 2C, χ^2^ = 0.20, *P* = 0.651). However, the trend suggested that right-sided disease may have poorer survival results.

## Discussion

Both PAOD and ABI are prognostic factors for predicting subsequent all-cause and CV mortality in the general population [7, 16, 17] as well as in HD patients [8, 9, 18]. In our study, we successfully demonstrated that the site of PAOD, as defined by ABI findings, was another independent predictor for all-cause and CV mortality in CHD patients. After adjustment for risk factors in the multivariate analysis, patients with right-sided PAOD had a higher risk for all-cause and CV mortality, whereas patients with left-sided PAOD did not. The two hypotheses were postulated to explain this finding. One was that right-sided ABI values may mean more severe vascular stenosis when they were the same to left-sided ABI values. Zhang et al. had reported the mean right ankle systolic blood pressure was about 1.7 mmHg higher than left side in Chinese population[19]. Therefore, the same ABI value in both sides didn’t mean the same atherosclerosis extent. The other possible explanation was that right-side disability may lead to a poorer prognosis because the general population is right-side dominant. Fig 2 shows a trend that right-side dominant ABI values had poorer survival outcomes in all patients and patients with/without PAOD. In fact, patients with right-sided PAOD also had higher calcium—phosphate product levels and lower Kt/v values, which suggested that this finding may be associated with poor dialysis adequacy and worse systemic calcium deposition. Therefore, to increase the dialysis dose and to control the serum calcium and phosphate levels in patients with CHD could prevent the developing of right-sided PAOD and delay the progression of vascular stenosis.

Cardiomegaly was higher in the group with abnormal ABI values on both sides. This finding was reasonable as some evidence had suggested that cardiomegaly was related to advanced age and malnutrition [20, 21]. Our data show that bilateral PAOD patients were older and had lower serum albumin levels. Serum calcium—phosphate product also lost its predictive power for all-cause mortality after adjusting for prognostic factors. This suggests that its impact might be also mediated through systemic arterial problems [22–24]. A low level of serum albumin has been a predictor for the poor prognosis of HD patients [8, 24–26].

The previously-identified risk factors for HD patients with PAOD included advanced age, DM, a history of CVD, smoking history, low diastolic BP, low pulse pressure, and low albumin levels [8, 27]; most were confirmed in our univariate analysis. However, several risk factors lost their significance in the multi-variate analysis. For example, although DM was a predictor of all-cause and CV mortality in our crude analysis, it was not significant in the multivariate analysis. The impact of DM on the multifactorial events that included vascular atherosclerosis and calcification was attributed to this phenomenon [8]. A CTR >0.5 was significantly related to the high mortality rate in our CHD patients and in diabetic HD patients [20]. However, Bohn et al failed to demonstrate CTR as a predictor of mortality in CHD patients [28]. Therefore, the clinical implications of CTR in HD patients for prediction of mortality are still controversial.

Our study has some limitations. First, it was single-center study, which might not be generalizable to all HD populations. Second, the patient population was small; nevertheless, this concern may be trivial because there was a significant relationship between PAOD site and mortality. Third, the gold-standard method for detecting PAOD is Doppler but we adopted ABI method to access the diagnosis of PAOD. Sometime, ABI method would lead to pseudomorphic normal when stenosis appeared in both arm and legs. However, ABI method had been extensively validated to be a reliable, and non-invasive tool for the diagnosis of PAOD[11, 29]. Finally, we only used baseline covariates to predict mortality. This may have resulted in biased estimates when the related variable was a time-varying predictor. However, there were two strengths in our current study. First, our follow-up duration was adequate to obtain sufficient numbers of deaths. Second, several well-established confounding factors related to dialysis therapy were adjusted.

To the best of our knowledge, this is first study that demonstrated an association between the site of PAOD and all-cause and CV mortality in CHD patients. The different findings on the site of PAOD may be attributed to the level of dialysis adequacy and calcium phosphate precipitation, which may contribute to vascular atherosclerosis and calcification. However, large-scale multicenter studies will be needed to verify our findings.

## Conclusion

The site of PAOD has been associated with all-cause and CV mortality in the Taiwanese HD population. We also found that right-side dominant PAOD was an independent predictor for mortality in HD patients independent of other predictors. We suggest routine screening for PAOD in HD patients with noninvasive ABI and that physicians should be vigilant for HD patients with abnormal ABI values, especially on the right side.

## Supporting Information

S1 Dataset The collected data in the study. (XLS)
